# Diffusion of global health norms through a national medical professional movement in the universal healthcare of Thailand

**DOI:** 10.3389/fpubh.2024.1249497

**Published:** 2024-03-07

**Authors:** Rangsan Sukhampha

**Affiliations:** ^1^Faculty of Sociology, Bielefeld University, Bielefeld, Germany; ^2^Department of Public Administration, Faculty of Humanities and Social Sciences, Valaya Alongkorn Rajabhat University, Pathum Thani, Thailand

**Keywords:** norm diffusion, global health, national medical professional movement, universal health coverage, Thailand

## Abstract

Commonly, research investigations on social policy reform primarily examine the national processes at the core of policy formation rather than considering their global context. Concerns are raised regarding the diffusion and influence of global health norms on Thai universal health coverage policymaking. The findings demonstrate that global health ideas and actors have an impact on national policymaking and that they can share ideas in a variety of ways, including glocalization, vernacularization, policy learning, and policy entrepreneur intervention, in setting the agenda for national universal health coverage. Global and universal health coverage (UHC) concepts have existed for decades; success would not be possible without the efforts of policy entrepreneurs such as the Rural Doctor Movement, who localize and vernacularize global concepts for implementation. These concepts must be compatible with the national and local sociopolitical contexts in which they exist. The Thai case contributed to a better understanding of the influences of global ideas and actors on transnational health policy transfer, as well as the intervention of the national medical professional movement as policy entrepreneurs in healthcare policymaking and policy change for equity in health.

## Highlights

- Commonly, research on social policy reform primarily examines the national processes at the core of policy formation rather than considering their global context.
- The article aims to establish a connection between the policy levels that are currently absent with a case study of global health diffusion in Thai universal health coverage (UHC).
- Examining the relationship between a national medical professional movement and the development of the Thai health system could offer valuable insights into how such a professional movement can enhance the national health system, particularly in relation to the interconnection of policy levels in global health.
- The implementation of global health policy at the national level requires the localization and vernacularization of global health ideas into a language that can be readily comprehended and appropriate for national and local audiences and contexts.
- A case study of Thailand’s UHC provides an understanding of how a national policy entrepreneur, such as a national medical professional movement, may serve as a catalyst and mediator to bridge the gap between global and national policy levels in the context of diffusion of global health norms.

## 1 Background

Prominent sociologists and political scientists have highlighted the significance of *“political opportunity structures”* (1), *“windows of opportunity*” (2), and *“critical junctures”* (3) for social and policy change. This article fills a gap in the literature on social policy change and policy entrepreneurs by describing a process by which policy entrepreneurs take strategic action that complements the current explanation of social policy change. In the presence of multiple independent or essentially independent streams of activities and participants, according to Kingdon (2), political entrepreneurs are most active in the policy stream, where they develop solutions for potential problems and advance them into the agenda-setting procedure. The purpose of this research is to challenge our understanding of conventional accounts that typically place national policy processes at the center by providing a missing lens on the formulation of the extensively discussed Thai universal health coverage policy. This study summarizes the complex discussion of the national medical professional movement’s contribution to the emergence of international concepts and players in Thailand’s health system reform in order to improve national health equity.

Global health concepts facilitate the transfer of health policy across state and national borders. It is possible to see how international actors transmit their intentions through the internalization of ideas and practices, as national medical professional movements frequently drive national public health reform. To comprehend the above-mentioned global phenomenon, it is necessary to investigate specific instances where international concepts and behaviors interact at the national and local levels. In this article, Thailand will serve as an example of how global health policy transfers, borrows, and adopts in the context of a medical professional movement for healthcare reform, specifically policymaking for universal health coverage (UHC). It is necessary to examine the democratization of Thailand concurrently with the country’s health system reform and UHC policymaking. Regarding McCargo (4), his article discussed the relationship between the monarchy and legitimacy crises in Thailand. The situation entailed the involvement in political affairs by King Bhumibol and his proxies, particularly former Prime Minister and President of the Privy Council, Prem Tinsulanonda and Anand Panyarachun. This included Dr. Prawase Wasi and his networks as well as his political philosophy, liberal royalist, and movement in the name of Rural Doctor Movement (RDM). According to Connors (5), in similar works with a focus on monarchy intervention, the term *“royal liberalism”* refers to the extra-bureaucratic networks that allow them to gain political influence and power through political networking. In some works about Thailand’s public health movement, Harris employs McCargo’s *network monarchy* theory as a lens for understanding Thailand’s UHC reform as the result of the tactical actions of a social network (4, 6).

In addition to the network monarchy theory, some works focus on the public health movement itself, such as Harris’s work on professional networks and the movement for universal healthcare policymaking, autonomous political networks, and health system reform, as well as the *“regulatory capture”* posed by developmental capture of the state and universal coverage policy (7). Sapyen’s work (8) examines how the RDM produced a novel strategy for a social movement that created the dual nature of the social movement in terms of non-governmental organizations (NGOs) within the governmental organization (GO), while retaining the nature of being a GO within NGOs. Vongtangswad’s work (9), which examines the conflict dynamics of the RDM and political transformation centered on framing and mobilization using three social movement frameworks: resource mobilization, framing process, and political opportunity structure theories, expands on this concept. The RDM implemented a dual autonomy structure for the healthcare system. A change in the political opportunity structure is a crucial factor that opens the door and enables rural doctors to modify their framing and mobilization to push for healthcare system reform. In practice, the movement collaborates with democratic and non-democratic political elites to promote public participation. As was previously mentioned, Nam (10) proposal for yet another important piece of work focuses on collaborating with government officials and NGOs to achieve universal health coverage (UHC), which rarely disregards external ideas and practices for influencing national health policymaking.

To institutionalize UHC in Thailand, medical doctor bureaucrats are incorporated into the Ministry of Public Health (MoPH) and affiliated with a specialized civil society group. As mentioned previously, the existing literature on Thailand’s health system reform and public health movement lacks global perspectives and connections due to the structure of health governance ideas and practices that are widely dispersed throughout the world. As a result, the study investigates how global and international organizations (IOs) influence policy transfers at the national level, as well as how developing countries influence the spread of health ideas from the Global South to the Global North. Based on the external and global health concepts presented in the Thai case study, the author describes how the national medical professional movement achieved national health equity.

The study’s overview is divided into the following parts. The first section gives a brief summary of the current scholarly research on global health norms to demonstrate the discipline’s discourse. Then, the following section provides examples of historical narratives that situate Thailand’s national medical professional movement within the context of the national development of the healthcare system. There, disclose the alteration in the movement’s framing and political opportunity structure and discuss their efforts to promote social change. The final section examines how global health concepts are incorporated into the Thai healthcare system. The concluding section explores how national medical professional movements and global idea diffusion influence national health policy.

## 2 How global health norms diffuse

Diffusion of global norms refers to the exchange of international ideas and practices at the international, regional, and national policy levels. It explains the global phenomenon of social policy in a globalized world in terms of its complexity, which is perhaps best illustrated by the movement of international norms and social policy across sectors. Swyngedouw’s work (11) popularized the term “glocalization,” which first appeared in a late-1980s Harvard Business Review article. Glocalization is a neologism formed by merging the terms “globalization” and “localization.” In this article, the term denotes the reciprocal interplay between global ideas and local actions, in contrast to the notion of vernacularization, which emphasizes the translation of global ideas into concepts and practices that are compatible with the way of doing things at the national and local levels (12). This involves adapting global ideas to accommodate the needs of citizens at the national and local policy levels. A few years later, he also coined the terms “glocal and glocalization” for multilevel policy analysis. Ilona Kickbusch’s work (13) expanded on Swyngedouw’s work to provide a further explanation of the term “glocal health,” which refers to the complex and interdependent interface between the theories and actions of global health actors and local efforts to promote health equity. The term “glocalization” was used in the context of reciprocity (14, 15) and can be applied to a variety of societal aspects. In the context of (global) health policy, glocalization can be used to analyze “glocal” idea transfer and how it spreads from the top down (from the Global North and global actors). In contrast, bottom-up concepts (from the Global South or national health concepts) can encourage international actors to adopt and refer to these local concepts in their (international) policy documents.

For instance, the 1948 establishment of WHO highlighted the transnational nature of health issues, and states admitted as members incorporated global initiatives into their national health policies (16). In the global social policy literature, global health governance investigates social policy issues deemed to have a global or transnational scope (17). This broader scope in terms of policies and actors participating in global healthcare generated diverse ideas and discourses that led to a contentious debate, such as in the case of IOs and global social governance (see Martens et al. (18)) regarding the “appropriated” (global) ideas for national implementation. According to Lee (19), the cognitive dimensions of globalization include understanding globalization and the contested construction of global ideas. In this instance, the thought processes are not limited to the Global North initiation, but the WHO and IOs, as global health actors, have cognitive authority over a specific field for the global idea and practice prescriptions. The approaches used for health, which are developed at the global level and directed by an IO such as WHO, are significant, but evidence of national translations and local actions should be the primary focus (20, 21).

Understanding the transfer of global health concepts from above can also be viewed as *Global Social Prescriptions* by IOs [see Deacon (22, 23) and Kaasch (24)] and its transfer of ideas from the Global North, as donor countries, to the Global South. Therefore, in the field of healthcare, it is necessary to illustrate the global health concept structure in IO debates. This topic is addressed in Deacon’s work (22), and global social policy consists of social policy prescriptions for national and supranational social policies concerning redistribution, regulation, and rights. In accordance with Kaasch’s work (24), she discusses the conceptualization of global social policy in the context of national social policy prescriptions regarding pensions and health systems. It reveals contention regarding concepts of the “positions war”. Moreover, Kaasch’s most recent work (25) proposes mapping the four major IOs in global health governance, focusing on the IO issue of the healthcare system: WHO, World Bank, ILO, and OECD, and conceptualizing the global health architecture based on competition and collaboration between them, as well as other social policies. Similarly, this article explores the conceptualization of global health actors as health policy prescriptions and attempts to transfer global health concepts to the national level. Not only Global Social Prescriptions by IOs but also donor countries from the Global North that give to recipient countries could be told how and what type of programs to operate.

Nitsan Chorev’s concept of *“Developmental Foreign Aid”* argues how foreign aid from the Global North shapes national and local public policy and national development of the Global South (26). On the other hand, in terms of diffusion from below, Chorev (27) argues in an article on *“reactive diffusion”* that ideas at the national level sometimes provide feedback and reshape global norms, and thus, health ideas could also be diffused from below. This does not imply that only global (North) ideas and actors can influence the Global South or less developed (in some dimensions) nations; initiatives from below can also inspire and serve as a model for the (global) policy papers of the IOs.

## 3 Global health norms diffusion through a national medical professional movement in Thailand

In order to comprehend the process by which global norms are diffused within the Thai health system, specifically in relation to UHC, it is essential to consider the contextual factors that influence the key actors responsible for making health policy choices in a given context. Witnesses in the Thai case have observed the movement of medical professionals advocating for the development of the health system throughout its history. Hence, examining the relationship between a national medical professional movement and the development of the Thai health system may offer valuable insights into how such a movement can enhance the national health system, particularly in relation to the interconnection of policy levels in global health. This section demonstrates the association between the evolution of the RDM’s framing and the development of the health system, which will be further explored in the following sections.

### 3.1 Foundation of public health in the early modern Thai state

Since the reign of Rama V, modern medicine in the contemporary Thai state has advanced gradually (1868–1910). In the first grant, healthcare policy was used to support national security and stability under bureaucratic political centralization in order to preserve absolute monarchy and the political influence of Bangkok’s elites at a time when Western nations were colonizing neighboring countries. Although Dr. E.A. Sturg, an American missionary, began construction on the first hospital in Phetchaburi Province in 1878, modern medicine did not reach a significant milestone until the establishment of Siriraj Hospital in 1886 and the Department of Nursing in 1888, both of which were affiliated with the Ministry of Education (28). Recognized as the beginning of these developments, the emergence of modern medicine and public health in Thailand during a variety of political conflicts is credited as the impetus for these advancements. King Rama V sought to reform and mobilize the nation in many areas where conservatives and aristocrats were in power by importing modern technologies and developmental ideas from the West (29). In order to increase the population and mobilize the labor force, regime stability was bolstered by lowering the mortality rate from cholera and plague. In the early stages of public health development, for instance, the Rockefeller Foundation assisted Thailand informally (1915) and formally (1921) in enhancing medical education (30). The existing establishment, however, faced the challenging waves of democratic, constitutional, and nationalist ideas that ultimately led to the 1932 revolution (31, 32). This had a substantial effect on the development and transformation of Thai public health.

The Cold War began in 1947 after an ideological dispute between the US and the USSR. The United States supported Field Marshal Plaek Phibunsongkhram’s coup d’état government’s fight against the communist threat in 1947 when Mao Zedong’s Chinese civil war was generally winning. As a result of the Cold War’s opening of both internal and external political opportunity structures, bureaucracy-centralized power continued to dominate Thai state politics. Field Marshal Sarit Thanarat, the coup d’état, and the traditionalist-conservative group usurped Field Marshal Plaek Phibulsongkhram’s authority in 1957, but the People’s Party’s (Khana Ratsadon) notions of democracy, constitutionalism, and nationalism would eventually fade away (33).

### 3.2 From public health for national security to public health for all

In 1942, the establishment of the MoPH gave rise to a new internal challenge force and political opportunity structure whose members were dissatisfied with the fundamental assumptions of the Thai state. Under the leadership of the dominant student movement, which is influenced by socialist and liberal democratic ideas, the internal movement reemerged (34). It was a cornerstone of the subsequent public health movement. Instead of emphasizing public health for national security, they shifted their focus to inequality, the concentration of power and resources, and public health for all, as opposed to public health for national security. The establishment of Thailand’s first faculty of public health, affiliated with Mahidol University in 1948, and the faculty of medicine at Ramathibodi Hospital in 1965, contributed to the subsequent changes in public health framing and movement (35).

In terms of health infrastructure, the Department of Medical Services began constructing provincial hospitals in 1947, in accordance with a government policy mandating the construction of provincial hospitals in all 72 provinces, as well as district-level health centers, between 1952 and 1957. It occurred during the administration of Field Marshal Plaek Phibunsongkhram (1948–1957), which could be considered the *“Golden Age of Building Provincial Hospitals.”* However, this would not have been possible without US Operation Mission (USOM) foreign aid and financial support for medical instruments. From 1951 to 1957, the US government provided $149 million in economic and financial support to the Thai government and $222 million in financial support to the Thai military (36). Bangkok’s port development, including transportation, trains, and roads, agriculture, education, irrigation, economic planning, public health development, and hospital expansion, has received economic and financial support.

### 3.3 Medical professional brain drain: state intervention and the establishment of the medical professional movement

In the latter half of the 19th century, in the 1970s, the Indo-China War, conflicts between communism and democracy across Southeast Asian nations, and the emergence of the “Dominos theory” intensified the fear of communist dominance over Thailand. In response, government policy aimed to alleviate poverty through rural development and improvements in health, education, and agricultural extension services. Thus, health, education, and agriculture became pillars of rural development and the reduction of poverty. During the Vietnam War in the 1970s, the US had to import medical workers from other nations, worsening the healthcare shortage and leading to brain drain in Thailand. Thai authorities responded with the first National Economic and Social Development Plan (NESDP) (1961–1966) (37). In 1965, the Office of the Civil Service Commission’s statistical data revealed that 52% of new doctoral students emigrated to work abroad. In response, the MoPH implemented a mandatory three-year rural health service placement policy in 1967, and the first graduate medical student began working in a rural area in 1972 (38). A group of medical students has protested against a three-year mandatory work requirement in rural areas, but ultimately they cannot stop the government’s intent.

The phenomenon of medical professional brain drain in the 1960s and 1970s, as well as state intervention in 1967, led to the implementation of a mandatory three-year rural health service placement policy for medical students. Most medical students have the opportunity to observe the lives of rural villagers, as rural doctors observe a large number of ill and impoverished patients. Some were forced to sell their farmland in order to afford medical care, resulting in catastrophic health expenditures and medical impoverishment. This event left them with such a bitter and painful experience that they hope to 1 day provide free medical care to the sick. This is one of the reasons for the creation of the RDM. The RDM’s role in achieving UHC has its origins in the rural doctors’ group, which first convened in April 1976 in response to a lack of physicians and difficult working conditions. Similar physicians come together to share, trade, and assist one another in various matters. A coalition of rural physicians was finally established at the first meeting held in Khao Yai, Pak Chong District, Nakhon Ratchasima Province. The Rural Doctor Federal was initially approved in 1976; after the event on October 6, 1976, the RDF had to cease operations. In 1978, the Rural Doctor Federal (RDF) was renamed the Rural Doctor Society (RDS) to avoid being monitored by the national security agency. This was done as the Rural Doctor Federal (RDF) was seen as an organization with a staunchly leftist ideology. According to the RDS’s goals, his statements are to be a hub for the exchange of ideas, the reinvention of services and academic knowledge, the enhancement of service efficiency, and the elevation of the status of practicing physicians in rural areas (39).

During that time, the RDM discussed the development of rural hospitals and the expansion of the RDS network in various regions. Later, in 1982, it became a legally registered NGO, the Rural Doctor Foundation (RDF), as a legal entity that could accumulate capital and mobilize resources, including social, intellectual, and financial sources (35). The RDF is primarily responsible for funding and publishing journals and brochures, as well as adhering to foundation establishment-related laws, whereas RDS is more flexible in terms of social activism (38). Individual members of the RDM have been maturing, being promoted to higher positions, and establishing a connection with the senior doctor in the MoPH. In certain instances, it appears that there are disagreements between doctors who serve the central government and those who work in rural areas. The recommendation of Prawase Wasi was to establish an academic platform that would facilitate communication between rural doctors who enjoy central authority in the MoPH and those who advocate for local change in rural areas. Dr. Prawase Wasi’s *“Triangle that Moves the Mountain”* has been used for decades as the RDM’s operational philosophy for running the platform. Since August 1986, the *Sampran Group Forum* has organized a monthly academic forum at Suan Sampran (Rose Garden) in Nakhon Pathom Province for (global) idea exchanges and academic meetings in public health services and administrations with the financial support of WHO Thailand (35).

### 3.4 A national medical professional movement and its vernacularization of UHC ideas

The vernacularization of UHC concepts into local practice is a challenging endeavor. Merry (40) defined vernacularization as a process of translating particular global ideas within context. Therefore, it is necessary to work through policy translators, here referring to policy entrepreneurs, in order to translate global ideas into concepts and practices that are compatible with the way of doing this at the national and local levels. As can be seen from the endeavor of policy entrepreneurs in the reformation of the Thai health system with accessible language and discursive programs, Sanguan Nittayaramphong’s work (35) discussed the power dynamics between the central MoPH and the local hospital. He worked at the Office of the Primary Health System Committee, which was embedded in a bureaucratic structure with a vertical management system and a lack of autonomy. A district hospital, on the other hand, offered different working conditions so that the doctor could play a leadership role with other agencies in the area, which is obviously related to RDM. In 1987, more committees were formed due to the RDM’s operational actions (41). Thai doctors in the US have collaborated with the American Medical Association to examine newborn and early neonatal mortality over the designated timeframe. The *“Running Campaign for No Smoking”* suggested a referendum in October 1987 to support the simultaneous adoption of the No-Smoking campaign and the *“Doctor Visit”* Program in a community hospital. During 1987–1988, the Foundation developed its first program, and the roles of the RDM (both the RDS and the RDF) became increasingly clear. The RDF is a funding source (e.g., social, intellectual, and financial sources), a formal organization, and a legal entity working in reconciliation and protection. The RDS is an informal organization that is not legally binding and has high mobility.

During 1985–1986, when Dr. Sanguan Nittayaramphong was chair of the RDF, the RDS engaged in a number of significant activities, including the promotion of UHC (41). When Dr. Sanguan worked in rural hospitals, however, the UHC initiative first began to take shape. During 1986–1988, he initiated an experimental research project in Khun Han District, Sisaket Province, with participants from universities, the MoPH, and local health workers. The objective was to observe health service operations and provide academic support for resolving operational issues. This study revealed that the majority of hospital patients presented with common illnesses and diseases and were able to care for themselves. As a result, the research suggests focusing directly on the community level and enhancing public health strategies. Villagers were urged to take better care of their own and their families’ health. In addition, it was determined that the prescribing practices of doctors at Khun Han Hospital were not excessive. Thus, comparing the average doses of drugs to the health centers’ drug use for the same medical treatment would help estimate the cost of services and enhance the health center’s capacity for treating common illnesses (35). In an effort to reform the healthcare system on a larger scale, the Ayutthaya Project, a prototype of UHC, was implemented between 1989 and 1994 as a result of a study of the conditions of rural hospitals. As agreed upon by the hospital and community, the experiment established a patient-centered integrated health system and collected a treatment fee of 70 baht per visit. After 2 years of operation, the Ayutthaya Community Medical Center at Wat Intharam continued to function normally with support from the state budget. Moreover, general hospitals, municipalities, and medical center personnel are pleased with their operations. This project’s success led to its expansion into a national initiative called the Health Care Reform Project, which was funded by the EU for approximately US$30,000 (35).

The ideas and experiences derived from the Ayutthaya Project stimulated reforms in public health financing, personnel, and referral systems and then expanded operations to other provinces. These ideas and practices in public policy driven by evidence-based health system research eventually morphed into an institution such as the Health Systems Research Institute (HSRI), in which the RDM played a significant role. The accumulation of evidence-based knowledge strengthens the RDM’s position to advance UHC when favorable conditions and policy windows of opportunity present themselves. In addition, the RDM expanded its network connections with various sectors of society, with Dr. Sanguan Nittayaramphong, a bureaucrat based at the MoPH, becoming involved in the Thai Rak Thai Party’s political sector and NGOs networking with the RDMs. These numerous networks motivate advocates to advocate for the attainment of UHC. In addition to the aforementioned healthcare research project, the RDM institutionalized the production of evidence-based knowledge to reform the health system. In 2001, the RDM established its institutional networks, such as the International Health Policy Program (IHPP), an international platform (42), and HSRI, to enhance health policy and systems research capability and international health policy development (43).

### 3.5 The window of opportunity opening for universal health coverage implementation

As stated, to comprehend Thailand’s health system reform and UHC policymaking, one must examine democratization alongside the sociopolitical contexts in which they are situated. Even though UHC ideas have been discussed in medical professional circles for decades, as noted in the preceding section, the national sociopolitical context and windows of opportunity for advancing these ideas as a vital national health policy agenda are currently obstructed. The contribution of the windows of opportunity to policymaking has been emphasized by a well-known public policy scholar, Kingdon (2). And in this instance, it could imply an understanding of the policy transfer of existing (global) health concepts elsewhere, such as global health concepts from the Global North and IOs and/or best practices from the Global South. This can be incorporated into national health policy, the internal context of which allows for national adoption.

In the late 1990s, after the political turmoil and tragedy of the so-called *“Black May Event,”* a protest against the government of General Suchinda Kraprayoon in Bangkok, and the bloody military crackdown that followed in 1992 [see McCargo (44) and Englehart (45)]. In addition, the 1997 Asian financial crisis that originated in Thailand, known as “Tom Yam Kung” (46), infuriated the public and prompted a call for a better quality of life for all as well as public participation in policymaking on multiple levels. A call for health system reform and UHC has been raised by the RDF as a political agenda, and its policy adoption by a political party, as it is fundamental to quality of life, could extend to another aspect of inequality reduction. This resulted in the opening of the social and political atmosphere, a juncture where all streams (problems, politics, and policy) converged to implement the previously conceived ideas.

Despite the fact that the Thai health system has undergone a significant transformation since the 1970s, when the government’s commitment to primary healthcare as a UHC foundation laid the groundwork for this transformation. This began in 1977 during the 4th five-year National Economic and Social Development Plan (NESDP) (1977–1981), which initially emphasized primary healthcare and investment in the health infrastructure at the local level, particularly in the 1990s at the district and sub-district levels (in the 2000s). Additionally, deliberate policies expanded the internal training of the health staff and spread to underserved areas and populations (47). After four decades of health infrastructure development and three decades of designing and implementing numerous financial risk protection schemes prior to the launch of universal health coverage, pilot phases were conducted in 2001, and four major public health insurance schemes covered four major population groups. 1978’s Civil Servant Medical Benefit Scheme (CSMBS) insured roughly 9 percent of the population. Approximately 7% of the population was covered by a Social Security Scheme (SSS) for formal sector workers in 1991. A 1983 subsidized voluntary health card (VHC) program covered 28% of the population, and a 1975 low-income card (LIC) program covered 23%. Only 2% of the population had private insurance, whereas approximately 30% did not (48).

Access to healthcare does not cover the entire population and tends to cause difficulties for rural doctors in rural hospitals. The Constitution of 1997 paved the way for the first election to be held. In December 1999, Dr. Sanguan Nittayaramphong drafted a yellow book describing UHC in terms of background, feasibility, and the budget required to achieve approximately baht 30,000 million (34). The only political party interested in his proposed UHC policy was Thaksin Shinawatra, the new leader of the Thai Rak Thai Party. Dr. Prommin Lertsuridej and Surapong Suebwonglee, as well as highly skilled doctors such as Dr. Viroj Tangcharoensathien, who was a member of the committee for the creation of a capitation system in the Social Security Scheme (SSS) in 1990, comprised his team of doctors who wielded power and played a crucial role in the party. SSS was distinct from the CSBMS’s fee-for-service system, which was later developed into a social security program with an initial copayment rate of 700 baht per person per year.

The political climate and the Constitution of 1997 became policy windows for UHC at that time. The Constitution herein offers a political framework that cultivates a robust government and facilitates efficient leadership. Following the implementation of political reform, a significant opportunity arose to advance comprehensive policies and address the pressing need for citizens’ rights. Consequently, all relevant parties recognized the crucial significance of lobbying for policies inside the public sector. As per the provisions outlined in Sections 52 and 82 of the Constitution of 1997, it was mandated that the state had an obligation to ensure that its citizens were granted fundamental access to medical care. The RDM, academics, and affiliates are gathering alliances to advance the UHC, while the health organization network has actively driven the proposal. The Thai Rak Thai Party has officially embraced the Universal Health Coverage (UHC) concept, which was among the nine campaign themes for the House of Representatives election in 2001. In order to engage in electoral campaigns, a catchphrase, namely *“30 baht for all treatments,”* was introduced. This particular slogan, which is more memorable than the conventional term *“Universal Health Coverage (UHC),”* serves as a political tactic. Subsequent to his successful election in 2001, Thaksin Shinawatra proceeded to unveil the government’s policy statement on February 26, 2001. This statement encompassed a range of programs, comprising immediate measures, economic strategies, initiatives to enhance revenue generation, policies pertaining to foreign commerce, and economic policies. UHC is one of nine policies that must be implemented immediately so that the state can reduce the overall expenditure on individual healthcare to a maximum of 30 baht per visit (equivalent to one dollar) and ensure that everyone has equitable access to healthcare. Therefore, the government has partnered with Mongkol Na Songkhla, a like-minded doctor based at the MoPH, as the Permanent Secretary of the RDM (10). Thus, the momentum for implementing the UHC initiative has increased significantly. According to Harris (7), the Thai case draws attention to bureaucrats’ ability to create policies in the absence of legislation and implement the policy as a pilot project before it becomes law, which in developed nations would be a laughable impossibility. The pilot phase’s UHC system was not implemented in six provinces until April 2001, was extended to fifteen additional provinces in June 2001, and was implemented nationwide on 1 April 2002.

However, advocating for and implementing the UHC agenda resulted in significant institutional changes and created political tensions within the MoPH by establishing NHSO as a quasi-governmental organization with a substantial budget in the health sector. This resulted in internal pressure and opposition from health personnel, who claimed that the UHC posed a long-term threat to the public health system. These healthcare workers insisted that the UHC created an overburdened workload and a disorganized implementation; as a symbolic protest against the system, *“Black Arm Doctors”* donned black armbands. The two competing ideas appear to constantly collide. According to Dr. Surapong Suebwonglee, achieving UHC is not an immediate objective; providing access to healthcare for 30 baths for all treatments is insufficient. Therefore, the national financing reform for public health must be reallocated. The public finance allocation for MOPH hospitals shifted from a supply-side allocation to a demand-side allocation with *per capita* budgeting (49). Moreover, the payroll system was centralized to ensure that healthcare workers continued to be paid on time.

Thailand achieved UHC in 2002 (pilot phase in 2001) and provided three main categories of Thailand’s Health System: the UCS covered 48.8 million people (about twice the population of Texas) and accounted for 70% of the Thai population; the Civil Servant Medical Benefit Scheme (CSMBS) covered 4.97 million and accounted for 10% of the population that were government employees, retirees, and dependents; and the Social Security Scheme (SSS) covered 13.09 million private-sector employees and their dependents (47). Members of the rural doctors’ network have founded or led executive-level positions in government, civil society, international organizations, academic institutions, and forums of national political significance. Harris’s work (6) introduces the term *“autonomous political networks”* to describe the RDM Network on the basis of the roles of Prawase Wasi and Sampran Forum as centers of the medical professional movement. Currently, the RDM is influential in Thailand’s health system reform. Despite being fragmented and embedded in multiple quasi-governmental organizations (QGOs), shared ideas and discourses have been institutionalized, i.e., NHSO, HSRI, IHPP, and Thai Health Promotion Foundation (ThaiHealth). Each QGO has also established its own network of NGOs in order to receive funding for the operation of the healthcare programs they support. Concurrently, the establishment of QGOs in the healthcare sector generated points of contention within the MoPH in terms of how public health would be reformed in terms of ideas and discourses, treatment and hospital extension focus, or disease protection focus. A purchaser-provider split, for instance, has undermined the central power of MoPH in terms of financial allocations, as purchasing power and the majority of funds have been allocated to QGOs, particularly NHSO, which plays the leading role as UHC purchaser (50).

## 4 Internalization of global health ideas in the Thai healthcare context

### 4.1 Global health ideas transfer: primary healthcare as the foundation of UHC

Regarding transnational policy transfer within the Thai UHC, I trace the formation of ideas for health system reform in the country alongside the RDM framing and reform prior to the establishment of the movement. This section examines the transfer of health policies from other nations, including community healthcare concepts from China that were developed prior to the WHO health policy recommendation on primary healthcare. Even though China did not attend the Alma-Ata conference because it was invading Vietnam, its ideas regarding community healthcare have influenced the conference’s outcomes (51). Prior to the 1978 Alma-Ata Declaration on primary healthcare, which the Soviet Union supported by hosting the conference, the community played a significant role in healthcare as a collective action throughout human history. The current local actors of PHC, the so-called *Community Health Workers (CHWs)*, can be traced back to Chinese Barefoot Doctors, the former *“Soviet Rural Feldsher”* and *“Thailand’s Village Health Volunteers (VHVs)”* (52, 53). A narrative about a local doctor responsible for the care of people in remote rural areas who employs local knowledge and traditional healing techniques with herbs and folk medicines has existed for centuries. In the modern state, however, the actions of rural doctors pertinent to political movements and social change are only available as historical records (54). The primary purpose of barefoot doctors was to promote hygiene, disease prevention, basic healthcare, family planning, and the treatment of common ailments (55). Throughout human history, transnationalization, both tangible and intangible, has occurred across national borders. This accelerates the spread of norms and ideas in various dimensions, including healthcare ideas, across international borders, from global health actors such as WHO to nation-states, and from nation-state to nation-state. In Thailand, the WHO, as a global actor, first introduced the PHC concept in northern Thailand as a pilot project; the first projects were *the Phitsanulok Project* (1966–1968) and *the Saraphi Project* (1968–1971) (56). South–South relations can be observed in Thailand during the late 1950s malaria epidemic, which resulted in an increase in the demand for health workers to combat the epidemic. Due to a lack of medical personnel providing healthcare services, community health volunteers were trained between 1961 and 1962 to combat the epidemic and eradicate malaria.

In fact, the People’s Liberation Army of Thailand, which was active from the 1940s to the 1990s, was the originator of VHVs (57). During the administration of Kriangsak Chamanan, the 4th National Economic and Social Development Plan (1977–1981) clarified the status of VHVs. In this way, VHVs played a significant role in the Cold War mass struggle; many people, including villagers, intellectuals, and doctors who were members of the People’s Liberation Army of Thailand fighting against the central government, fled into hiding to avoid state repression. Consequently, the term *“barefoot doctor”* was derived from the characteristics of barefoot healthcare workers in the field, such as those in the People’s Liberation Army of Thailand. In conclusion, the *“Chinese barefoot doctor”* concept, which has crossed national borders in large numbers, has influenced the VHV concept in Thailand. In addition, the success of barefoot doctors in providing access to medical care for rural people in remote areas inspired the 1978 World Health Organization Conference on Primary Healthcare, where it was finally decided to release the Alma-Ata Declaration (58). Notwithstanding, these global health concepts have a significant impact on the adoption of PHC at the national level throughout the world, and PHC is the foundation for UHC achievement, as seen in the case of Thailand and other LMICs.

### 4.2 A national medical professional movement: policy entrepreneurs as translators

The term *“professional movement”* is used in Harris (59) work to examine the effort to expand access to healthcare and AIDS medication in Thailand, Brazil, and South Africa. Harris referred to the RDM in Thailand as “progressive doctors” who played a vital role in UHC policymaking. In Brazil, a comparable movement of medical professionals concerned with public health played crucial roles in drafting legislation in the Health Ministry and promoting programs to provide healthcare to the masses and hold the state accountable. However, South Africa only made incremental progress because the government actively obstructed professional movements seeking more radical reform. In Thailand, the Rural Doctor Society, a national medical professional movement and institutional entrepreneur, does not stand alone; it also engages with global health concepts derived from the WHO, ILO, and the World Bank. Even though these global actors did not directly influence the implementation of UHC in the countries where they were involved, the health minister requested the assistance of the WHO and the ILO to organize a technical seminar that gave the reform strong support. In addition, the EU financed the healthcare reform program and provided technical and research skills to a large number of medical doctors through training programs at medical schools such as Antwerp University and the London School of Hygiene and Tropical Medicine (56). As stated previously, this demonstrates how a national medical professional movement in certain countries has been influenced by global health concepts and practices in order to advance national health reform.

From their ideas, beliefs, and participation in social cultivation processes, it is possible to determine how a national medical professional movement as policy entrepreneurs has been operating. This can be deduced from the publications and media they have disseminated, which demonstrate their explicit knowledge. Numerous important policy entrepreneurs engaged in health policy change are worthy of mention in this instance; for the development of UHC, Sanguan Nittayaramphong and his colleagues are essential. The critical role that medical doctor bureaucrats embedded in the Ministry of Public Health and affiliated with professional groups of civil society play in Thailand’s efforts to institutionalize UHC. The RDM reveals a dual nature, with NGOs embedded within governmental organizations (GOs) and the nature of GOs maintained within NGOs. This dual nature enables them to mobilize resources to advance UHC, including civil society resources funded by foreign sources, political and bureaucratic resources, and symbolic resources from international organizations. To advance UHC in the 1990s, the RDM is a collection of civil society resources funded by foreign sources. The rural doctor network pushed an important EU-funded project, the so-called Health Care Reform Project, to help mobilize public health reform in terms of funding, personnel, and referral systems and then expanded operations to other provinces.

In addition, the RDM has access to resources for reform mobilization, such as symbolic resources from IOs as reference power and the creation of debate against reform resistance, which may lose their advantages if the current system is altered. In this context, a discussion and argument for Thailand’s UHC initiative can be viewed as a contestation of global health ideas in which global actors and ideas are at odds. It matters which services are covered and how they are funded, managed, and delivered to low-income and vulnerable populations. In 2001, when Thailand’s GNI *per capita* was approximately $1,900, WHO voices strongly supported UHC taxation implementation. Based on the evidence, UHC can reduce catastrophic health expenditures and medical impoverishment and increase access to healthcare for all, even though Thailand had only recently recovered from the financial crisis of 1997 (47).

On the other hand, the World Bank strongly critiqued and warned that the country would eventually face a public financial collapse and bankrupt public hospitals. The bankrupt financing system will dilute healthcare quality if Thailand is still stubborn about doing so. In addition to the World Bank stance, in 2000, before the new election in 2001, Dr. Sanguan Nittayarumphong handed in policy proposals and drafted the National Health Security Act to the Democrat Party, but Chuan Leekpai, Prime Minister at that time, did not see the possibility of succeeding. And he rejected Dr. Sanguan’s proposal, saying, “Not ready, not enough budget.” This is often the golden paragraph of the *“Democrat Party”* (Quoted in an interview with the media by Dr. Kriengsak Watcharanukunkiat), former President of the RDF (60). Their stance still insisted on the same even after defeating the election and switching to the position party. Thus, the influence of ideas and discourse in Thailand’s health system reform created a better understanding of how global ideas have shaped national health policy in one way or another.

## 5 Perspectives on the Thai UHC in the global-national context

Many countries have been faced with transnational health issues that demand cooperation beyond the state border, and this phenomenon opens an opportunity for global and transnational actors to play an immense role in coping with challenges. This article has explored the linkage between the external influences of global health ideas and the internal professional movement in achieving UHC policy in Thailand and discusses vital global ideas and practices that the RDM in Thailand considers mobilizing health system reform for equity in health. The global trends in the UHC were endorsed in the SDGs for global achievement by 2030, especially in low-income countries.

To the best of our knowledge of transnational policy transfer, this study attempts to fill the knowledge gap in the field and identify mechanisms and communication channels for multilevel policy transfer. The study examines how discussions on global health ideas influence national health system reform and policymaking. This highlights the impact and role of global ideas on national professional movements, such as the RDM in Thailand, in mobilizing health system reform. The findings reveal that global health ideas transfer through instruments, mechanisms, and communication channels in multilevel contexts from the global to the national, or vice versa. As can be seen from Thailand’s leadership in the global health movement for UHC: as a well-documented, inspiring, and shared case study success in the 2005 resolution for UHC at the WHA, as a founding member of the ministerial group of Foreign Policy for Global Health, the UNGA UHC Declaration in 2012, the 2015 SDG inclusion of UHC targets, the 2018 and 2023 High-Level Meeting on UHC in New York (61), and as the current Chair of the Foreign Policy and Global Health (FPGH) Initiative (62). The Thai UHC presently encompasses almost the entire population, surpassing the SDG 3.8 pledge made at the United Nations General Assembly in September 2015. Thailand, as the pioneer in UHC among low- and middle-income countries (LMICs), prioritizes UHC in government, foreign affairs, and health agendas. The country has been progressively enhancing its monitoring and evaluation (M&E) platform in a more detailed manner than the SDG Indicators 3.8.1 and 3.8.2. Thailand utilizes multiple data sources to track three dimensions of UHC (population coverage, service coverage, and financial risk protection) as well as four key sources (national surveys, health facility and administrative data, specific disease registries, and research). Each source offers distinct advantages and is used simultaneously to enhance the information provided by the others. The experiences of Thailand can offer valuable insights for LMICs on enhancing their M&E systems for achieving UHC (63).

Furthermore, global health idea transfer can occur to a greater extent from IOs to national states vertically and from national states to national states horizontally. Most literature on Thailand’s health system reform often focuses on national processes in the first place (7, 10, 64–66). In practice, achieving equity in health at a national level could not be possible without transnational or global health idea transfer, and in reverse, national best practices can inspire global actors to adopt national ideas into their policy papers, such as the national universal health coverage achievement in Thailand.

Regarding health idea translation at the national level, the RDM and individual agencies, as policy entrepreneurs, translate external or global ideas and practices for their movement and mobilization. Some scholars investigate policy transfer and transmission among developed countries and/or from developed countries to the developing world (67). This study exposes the transnational policy transfer of ideas and practices across actors, such as policy transfer from IOs to developing countries or the reverse, from developing countries to developing countries themselves. These global ideas are related to the national context of health system reform and policy agenda-setting, which are the architecture of arguments in global health ideas (25), glocalization (13), and vernacularization (68, 69). This includes universal health coverage and its debates, primary healthcare, and health idea transfer, including the roles of the medical profession and associations as mediators for health idea transfer. Global and national linkage exists in one way or another in different forms, such as the international forum and financial aid for health infrastructure and project development.

On the other hand, internal movement can be explored in a macro-level context (such as the institutional and structural faces of context) and a micro-level context (such as agency-level enabling conditions) [see the similar approach of Bakir and Jarvis (70)]. The global ideas and practices vernacularized or transferred to national health reform must be adjusted and suitable for a particular national context (12). Prominent scholars have pointed to the importance of changes in *“political opportunity structures”* (1), *“windows of opportunity”* (2), and *“critical junctures”* (3) to social and policy reform and change, with the roles of policy entrepreneurs in the agenda-setting that may result in high impact policy and social change. In this study, this policy entrepreneur actively engaged in health system reform in the policymaking process situated in problems, policy, and politics streams. In a similar vein, without policy, entrepreneurs (individuals and sometimes small groups of people) play a role in the public policy process as the agency for translating ideas into action, and social policy reform and change might not be possible (71). In a Thai case, the *Ayutthaya Project* provided fundamental findings for further policy extension of the recent UHC. The 1992 Black May and the 1997 financial crisis as windows of opportunity contributed to the election of Thaksin Shinawatra with the *“30 baht for all treatment*” healthcare scheme, which all proved foundational for establishing UHC.

However, in terms of execution, a policy entrepreneur, such as savvy bureaucrats, social movements, or medical professional movements, plays a crucial and advantageous role in leveraging a universal platform and emphasizes that a national medical professional movement is advantageous for the localization and implementation of global health ideas. A national medical professional movement faces numerous challenges in terms of achieving unity, managing internal group dynamics, and addressing fragmentation within the MoPH. Drawing from the Thai experiences in health system reform, it can be inferred that the successful implementation of global health ideas at both national and local levels requires a policy entrepreneur (e.g., professional movement) who can effectively connect the various components of policy, politics, and problem-solving. This entails localizing and vernacularizing global ideas to align with the specific national and local contexts.

## 6 Conclusion

The 2015 SDGs support UHC as the global development agenda for global achievement by 2030, and it is anticipated that UHC will help shape national health agendas and policymaking. In order to implement Thailand’s policy of universal health coverage, this study examines how international and external health ideas affect national health policymaking. The findings demonstrate that the UHC requires the participation of the national medical professional movement in other countries, particularly in low- and middle-income countries (LMICs), to drive the initiative and make concerted efforts (such as intervention by policy entrepreneurs, the vernacularization of ideas for policymakers, and the translation of these ideas into policy implementation). Based on the Thai experience, for instance, the RDM plays the role of an agency that adapts concepts of global health to institutionalize them and incorporates them into the national knowledge production process for evidence-based policy when the political opportunity structure is highly flexible. In conclusion, the Thai case helped to clarify how global health actors and ideas can have an impact on national policymaking with the help of the national professional movement, which acts as a policy entrepreneur in implementing transnational ideas and practices. To accomplish initiatives that other nations can learn from, this includes collaborating with numerous groups and governing networks.

## Author’s note

RS is currently a doctoral researcher at the Faculty of Sociology at Bielefeld University in Germany and also a faculty member of the Department of Public Administration at Valaya Alongkorn Rajabhat University in Thailand. His interests are in public administration, political sciences, global and transnational social policy, health policy and governance, international relations, and Southeast Asian and ASEAN studies.

## Author contributions

The author confirms being the sole contributor of this work and has approved it for publication.
